# Artificial intelligence applied to diabetes complications: a bibliometric analysis

**DOI:** 10.3389/frai.2025.1455341

**Published:** 2025-01-31

**Authors:** Yukun Tao, Jinzheng Hou, Guangxin Zhou, Da Zhang

**Affiliations:** Department of Endocrinology, Air Force Medical Center, Air Force Medical University, Beijing, China

**Keywords:** artificial intelligence, diabetes complications, bibliometric analysis, deep learning, machine learning

## Abstract

**Background and aims:**

Artificial intelligence (AI)-driven medical assistive technology has been widely used in the diagnosis, treatment and prognosis of diabetes complications. Here we conduct a bibliometric analysis of scientific articles in the field of AI in diabetes complications to explore current research trends and cutting-edge hotspots.

**Methodology:**

On April 20, 2024, we collected and screened relevant articles published from 1988 to 2024 from PubMed. Based on bibliometric tools such as CiteSpace, Vosviewer and bibliometix, we construct knowledge maps to visualize literature information, including annual scientific production, authors, countries, institutions, journals, keywords and research hotspots.

**Results:**

A total of 935 articles meeting the criteria were collected and analyzed. The number of annual publications showed an upward trend. Raman, Rajiv published the most articles, and Webster, Dale R had the highest collaboration frequency. The United States, China, and India were the most productive countries. Scientific Reports was the journal with the most publications. The three most frequent diabetes complications were diabetic retinopathy, diabetic nephropathy, and diabetic foot. Machine learning, diabetic retinopathy, screening, deep learning, and diabetic foot are still being researched in 2024.

**Conclusion:**

Global AI research on diabetes complications is expected to increase further. The investigation of AI in diabetic retinopathy and diabetic foot will be the focus of research in the future.

## 1 Introduction

Diabetes is a global disease, and with the large-scale urbanization and aging population, the number of diabetic patients is rapidly increasing. According to the International Diabetes Federation Atlas, by 2045, the global prevalence of diabetes among the 20–79 age group will rise to 12.2% (783.2 million people) (Sun et al., 2022). Diabetes complications are the leading cause of death for diabetic patients, including diabetic retinopathy (DR), diabetic peripheral neuropathies (DPN), diabetic foot (DF), diabetic nephropathy (DN), diabetic cardiomyopathies, hyperglycemic hyperosmolar nonketotic coma, and diabetic ketoacidosis (DKA). As the number of affected individuals continues to rise, diabetes and its complications will consume substantial public health resources and pose numerous economic challenges.

The concept of artificial intelligence (AI) was first proposed in 1955 (Hamet and Tremblay, 2017). It is a highly complex discipline primarily composed of machine learning (ML), deep learning (DL), convolutional neural networks (CNNs), and recurrent neural networks. AI can assist computers in analyzing vast amounts of clinical data, enabling them to learn the most predictive features and establish predictive models, which aid in personalized treatment and improve diagnosis (Sarker, 2022; Jiang et al., 2017). In recent years, AI has been widely applied to the screening (Vaľková et al., 2024; Farahat et al., 2024), treatment and prediction (Kim et al., 2020; Shin et al., 2022) of diabetes complications. It is anticipated that AI-driven precision medicine will be developed in the future to predict and diagnose diabetes complications (Huang et al., 2023). However, there are still challenges in the future in terms of data standardization, interpretability of results, and generalizability to other scenarios (Spasić et al., 2014).

While numerous reviews have been published on the application of AI in diabetes complications, they primarily focus on the early diagnosis, treatment, and prediction of individual complications, such as diagnostic screening of DR (Bai et al., 2024), early detection and prediction of DF ulcers (Wu et al., 2024), and diagnosis and prognosis of DN (Dholariya et al., 2024). A search on PubMed revealed that bibliometric studies in this field are predominantly concentrated on DR (Poly et al., 2023; Wang et al., 2022; Xiao et al., 2023; Shao et al., 2022). Consequently, there is still a lack of systematic review and visualization analysis of AI research in diabetes complications as a whole. Based on this, we conducted a bibliometric analysis and review of literature on AI in diabetes complications to further understand the applications, trends, and future prospects of AI in predicting, diagnosing, and treating diabetes complications.

## 2 Materials and methods

### 2.1 Data sources

PubMed, as the most widely used database in the field of medicine, encompasses a vast array of medical literature covering a broad spectrum of knowledge. Moreover, it is freely accessible to all medical professionals. Therefore, we utilized PubMed as the database for conducting Mesh term searches and subsequently performed bibliometric analysis on the retrieved results.

### 2.2 Search strategy

**Figure 1:**
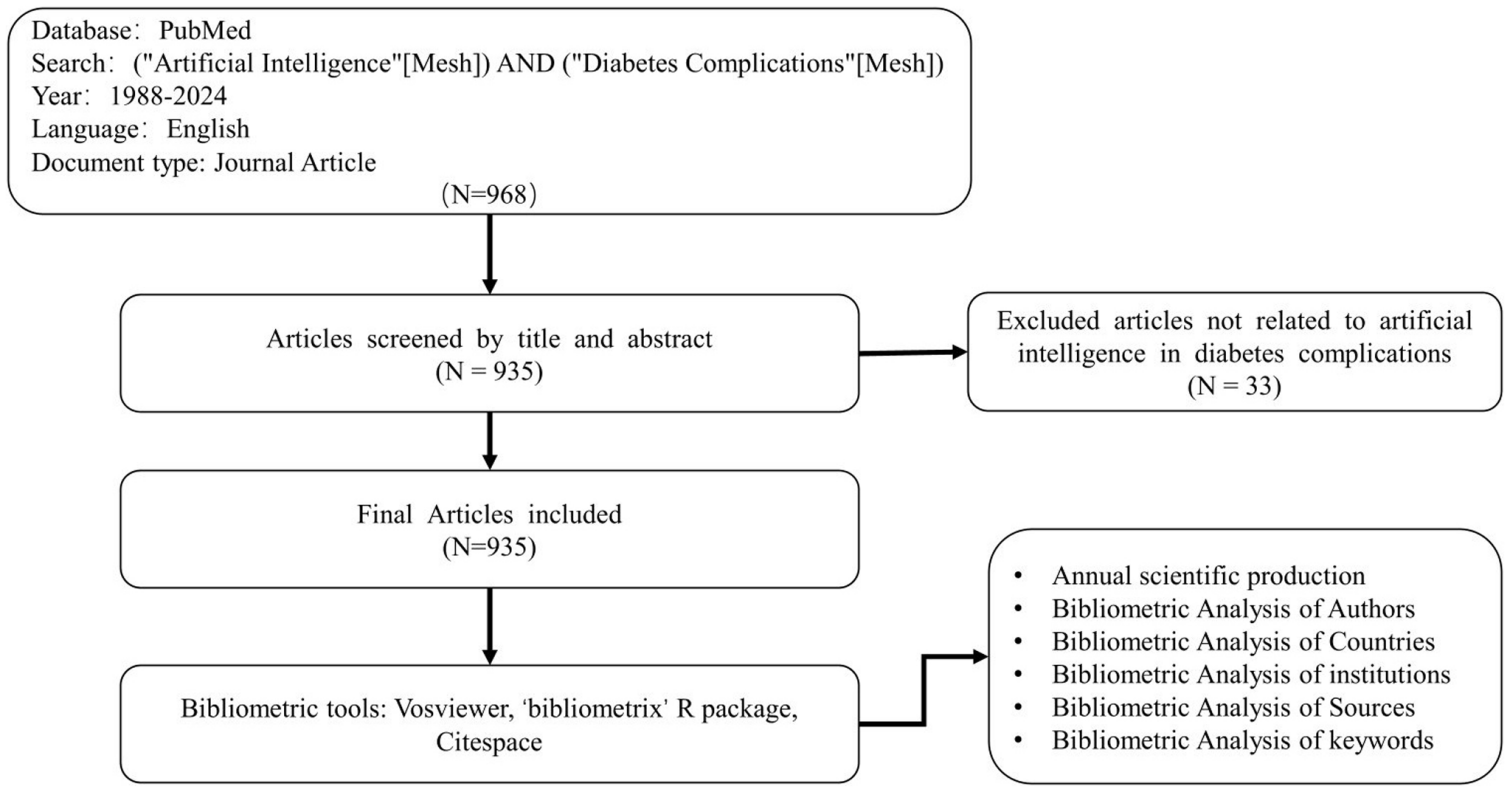
Search flow diagram. The diagram shows details selection criteria for AI related to diabetes complications from PubMed and bibliometric analyses.

On April 20, 2024, we downloaded the required literature data from the PubMed database using the following search strategy: (“Artificial Intelligence”[Mesh]) AND (“Diabetes Complications”[Mesh]). For our search, we opted for Mesh term searching. The subheadings under AI included: Computer Heuristics, Expert Systems, Fuzzy Logic, Knowledge Bases, Biological Ontologies, Gene Ontology, ML, DL, Supervised Machine Learning, Support Vector Machine, Unsupervised Machine Learning, Natural Language Processing, Neural Networks, Computer, Robotics, Sentiment Analysis. Similarly, the subheadings under Diabetes Complications included: Diabetic Angiopathies, DF, DR, Diabetic Cardiomyopathies, Diabetic Coma, Hyperglycemic Hyperosmolar Nonketotic Coma, DKA, DN, DPN, Fetal Macrosomia. To encompass a wider range of literature, we set the search timeframe from 1988 to 2024. The specific search strategy is outlined in Figure 1.

### 2.3 Research tools

Bibliometrics involves analyzing a large volume of literature in a specific field to gain an overall understanding of that domain. Pan et al. (2018) compared 10 bibliometric mapping software tools and the number of web of science papers mentioning these tools in the field in 2018. They found that CiteSpace was the most widely used, with a total of 78 papers, followed by Vosviewer, with 70 papers. Therefore, for this study, we selected CiteSpace and Vosviewer as analysis tools. Additionally, bibliometrix was also utilized, which is a software package in the R language capable of visualizing and analyzing retrieved literature.

### 2.4 Bibliometric analysis

A bibliometric analysis was conducted on 935 articles to provide insight into the research landscape of AI in diabetes complications. This analysis included annual scientific production, authors, countries, institutions, journals, keywords and research hotspots.

According to Price Law (De Solla Price, 1963): $m=0.749\times \sqrt{n_{\text{max}}}$ (m corresponds to minimum number of publications and n_max_ corresponds to the number of papers by the most prolific authors in a field), we can calculated the minimum number of publications for core authors in a field.

The growth rate of publications was calculated as follows: $G=\sqrt[{n}]{a_{1}/a_{2}}\times 100$ (where a_1_ refers to the number of publications in the most recent year, a_2_ refers to the number of publications in the first year, and n is the number of years) (García-Jaramillo et al., 2024).

Network analysis was used to examine the scientific, social, intellectual, and conceptual structures of scientific production in the field of interest (Omotehinwa, 2022). We conducted network analysis of authors, countries, institutions, and keywords in Vosviewer and CiteSpace. In Vosviewer, each node represents an object (an author/institution/keyword), and the size of the node indicates the frequency of occurrence, and the color of the node represents different clusters. The connections between nodes represent the level of collaboration among objects, referred to as total link strength (TLS) in bibliometric analysis. TLS is primarily determined by factors such as the number of co-authored publications, co-citations, or joint research projects. A stronger connection indicates a higher degree of collaboration or citation relationships. In CiteSpace, the concepts are consistent except for centrality. Centrality signifies the number of shortest paths passing through a node in a network. The purple circle surrounding a node represents the centrality value, with thicker purple circle indicating higher value. A higher centrality value indicates greater influence in communication among other nodes, suggesting higher prominence. Keywords with the strongest burst in citations was used to explore the continuity and variability of research hotspots in the application of AI in diabetes complications from 1988 to the present using CiteSpace.

All original data used in this research were sourced from the publicly accessible PubMed database, therefore no ethical approval was required.

## 3 Results

We categorized the retrieved 935 documents into four main classes based on different diabetes complications: DR (695 articles), DPN and DF(96 articles), DN(78 articles), other complications(66 articles).

### 3.1 Annual scientific production

**Figure 2:**
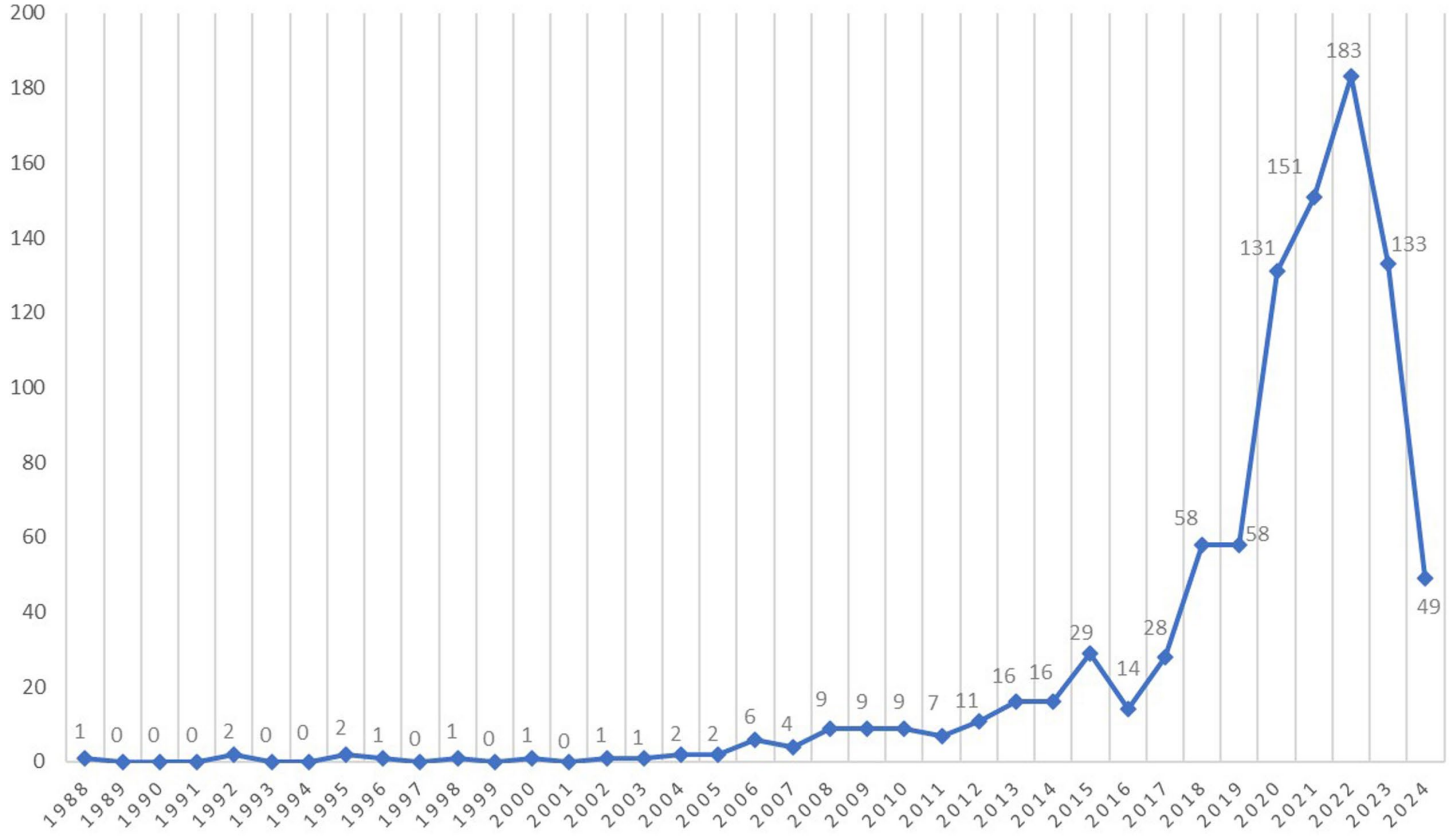
Line graph. The graph shows total number of publications per year in the field.

Based on the analysis of publications retrieved from the PubMed database regarding the application of AI in diabetes complications, a total of 935 articles were selected. These articles originated from 2,748 institutions and were authored by 4,757 authors from 70 countries. They were published across 249 journals. Figure 2 illustrates the temporal distribution of publications in the field of AI for diabetes complications. Overall, the research on the application of AI in diabetes complications began with the publication of the first paper by Wiener (1988) titled “SMR (simulating medical reasoning): an expert shell for non-AI experts” in 1988. The number of publications gradually increased after 2005, with a small growth rate. However, after 2016, there was a significant increase in publications. The number of publications remained consistently above 100 between 2020 and 2023, reaching its peak in 2022 with 183 publications. We calculated the growth rate of publications for this topic to be approximately 15% based on the number of articles published in 2023 as the most recent year. This indicates sustained interest among scholars in this research area, gradually establishing it as a new hotspot in the field of diabetes complications research.

### 3.2 Bibliometric analysis of authors

**Figure 3:**
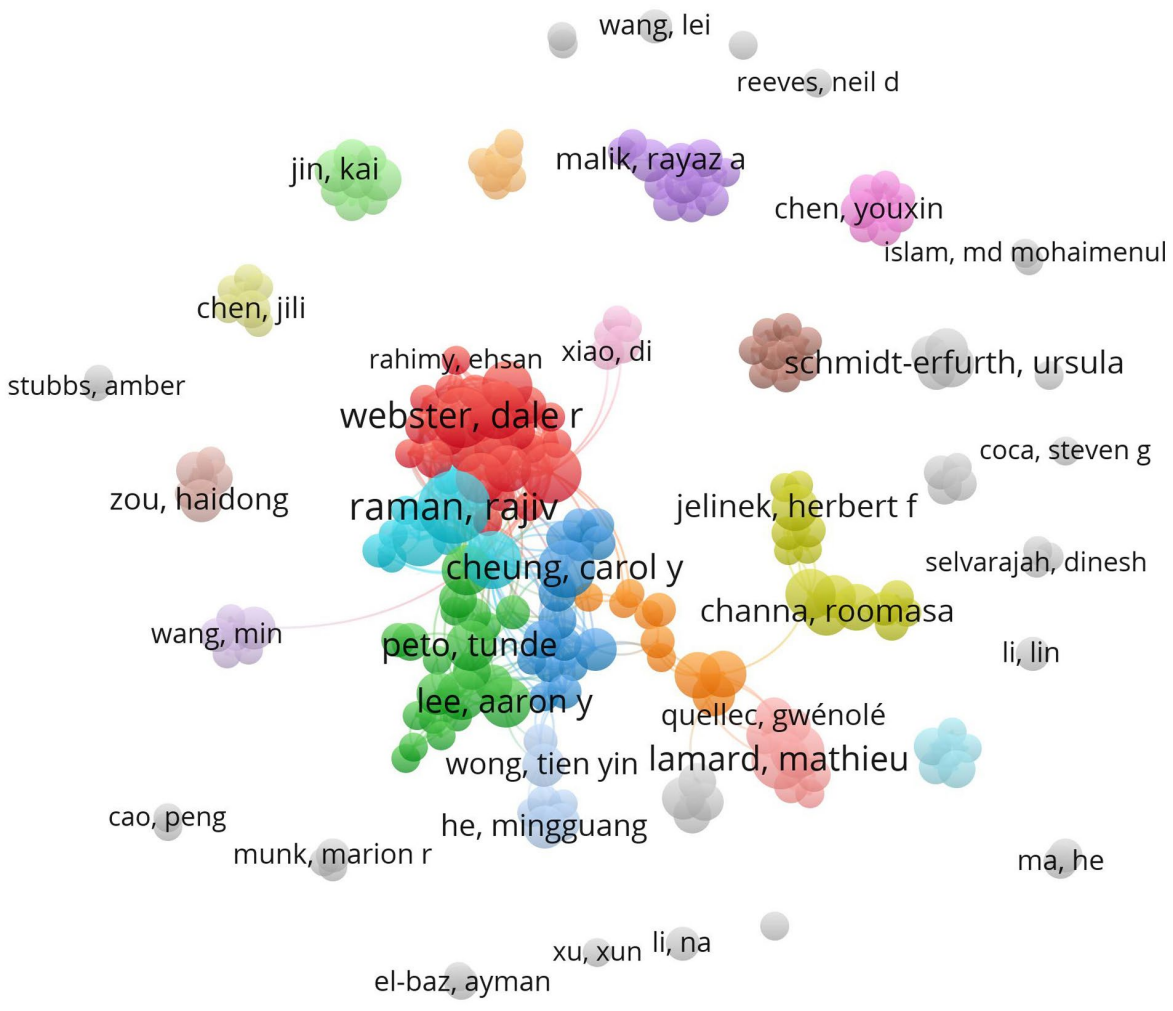
Co-authorship network map of core authors. Each node in the map represents a core author, and the size of the node corresponds to the author’s productivity (i.e., the number of publications). The lines connecting the nodes illustrate the collaboration between the authors, with the thickness of the links indicating the level of collaboration between them.

A total of 4,757 authors have published articles related to the research on AI in diabetes complications. Among them, 4,086 authors have published only one article, accounting for 85.9%, while 671 authors have published two or more articles, accounting for 14.1%. The minimum number of publications for core authors in this field is approximately 2.9. Authors who have published three or more articles (including three articles) are identified as core authors in this field, totaling 224 individuals. Figure 3 illustrates a co-authorship network map of core authors. Among these core authors, connections exist between some of them. Table 1 presents the top 16 productive authors and TLS. The top three most productive authors are Raman, Rajiv (publication = 15, TLS = 61), Webster, Dale R (publication = 11, TLS = 102), and Cuadros, Jorge (publication = 11, TLS = 68). The top three authors with the highest TLS are Webster, Dale R (publication = 11, TLS = 102), Peng, Lily (publication = 10, TLS = 93), and Corrado, Greg S (publication = 8, TLS = 76). It is noteworthy that Webster, Dale R has a significant number of publications and the tightest connections, indicating substantial academic contributions in this field and extensive collaboration with other authors.

**Table 1:** The top 16 productive authors and total link strength.

Rank	Authors	Publications	TLS	Avg. pub. year
1	Raman, Rajiv	15	61	2021
2	Webster, Dale R	11	102	2021
3	Cuadros, Jorge	11	68	2020
4	Peng, Lily	10	93	2021
5	Sivaprasad, Sobha	10	38	2021
6	Lamard, Mathieu	10	35	2015
7	Cheung, Carol Y	10	29	2022
8	Cochener, Béatrice	9	34	2014
9	Rajalakshmi, Ramachandran	9	20	2021
10	Corrado, Greg S	8	76	2021
11	Ruamviboonsuk, Paisan	8	67	2021
12	Keane, Pearse A	8	39	2022
13	Tufail, Adnan	8	33	2022
14	Abramoff, Michael D	8	17	2018
15	Lee, Aaron Y	8	15	2022
16	Peto, Tunde	8	10	2020

TLS, total link strength.

### 3.3 Bibliometric analysis of countries

**Figure 4:**
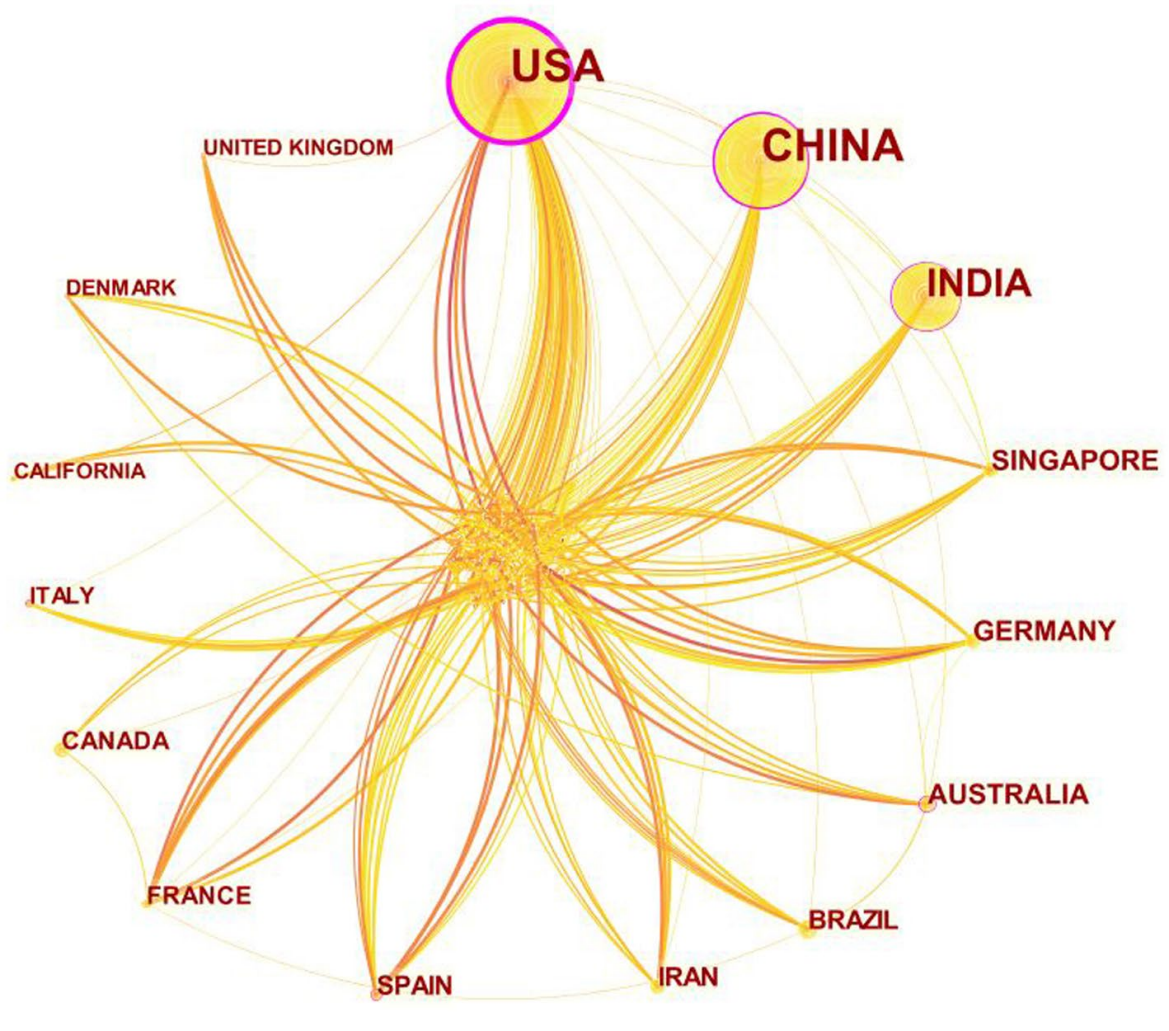
Cooperation of Countries that contributed to publications on the use of AI in diabetes complications from 1988 to 2024. Each yellow node represents a country. The yellow lines connecting the nodes illustrate the collaboration between the countries, with the thickness of the links indicating the level of collaboration between them. The purple circle surrounding a node represents the centrality value, with thicker purple circle indicating higher value.

The United States leads in publication output, with 200 articles published, accounting for 21.39% of the included research articles. China and India follow closely with 190 and 80 articles, respectively, placing second and third. Additionally, the United States has the highest centrality (0.86), followed by China (0.29) and Australia (0.16) (Figure 4; Table 2). The United States is considered the most influential country in this field. It’s noteworthy that although Australia ranks sixth in publication output, it ranks third in centrality, indicating that articles published by Australia have considerable influence.

**Table 2:** The top 10 productive countries and centrality.

Rank	Countries	Publications	Centrality	Year
1	USA	200	0.86	1992
2	China	190	0.29	2010
3	India	84	0.12	2009
4	Singapore	23	0.06	2008
5	Germany	22	0.08	2000
6	Australia	20	0.16	2007
7	Brazil	19	0.04	2012
8	Iran	18	0.05	2009
9	Spain	17	0.12	2008
10	France	15	0.06	1995

### 3.4 Bibliometric analysis of institutions

**Figure 5:**
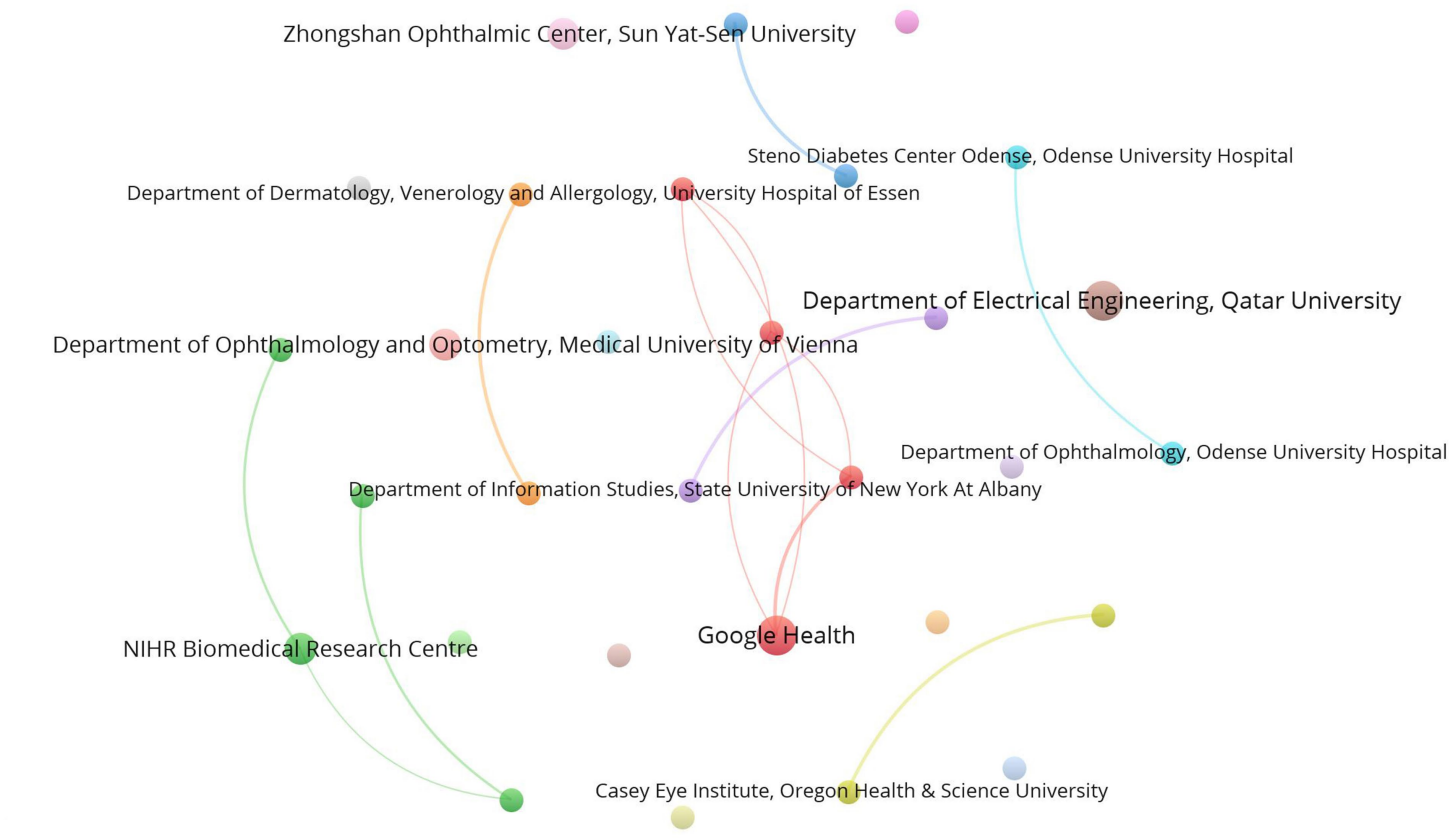
Co-authorship Network Map of Institutions. These 30 institutions have been divided into 19 clusters with 14 links and a total link strength of 27. Each node represents an institution, and its size indicates its productivity. The lines connecting the nodes depict their collaboration, and the thickness of the links represents the level of collaboration.

A total of 2,748 institutions worldwide have participated in research on AI in diabetes complications. 30 institutions have published more than 3 articles. It can be observed that there is relatively little collaboration among institutions, and the overall distribution of institutions is quite scattered from Figure 5. The largest institutional collaboration cluster is the red cluster centered around Google Health. Table 3 presents the top 10 productive institutions and TLS. The institutions with the highest number of publications are Google Health (5), Department of Electrical Engineering, Qatar University (5), NIHR Biomedical Research Centre (4), Department of Ophthalmology and Optometry, Medical University of Vienna (4), and Zhongshan Ophthalmic Center, Sun Yat-Sen University (4). The remaining 5 institutions have a publication output of 3 articles each. In terms of TLS, Google Health ranks first with a value of 5, while Qatar, Austria, China, and Singapore have a TLS of 0. The remaining institutions all have a TLS of 3. It is noteworthy that among the top 10 research institutions ranked by publication output, three institutions are from the United States, highlighting the dominant position of the United States in the field.

**Table 3:** The top 10 productive institutions and total link strength.

Rank	Institution	Countries	Publications	TLS	Avg. pub. Year
1	Google Health	USA	5	5	2022
2	Department of Electrical Engineering, Qatar University	Qatar	5	0	2022
3	NIHR Biomedical Research Centre	UK	4	3	2022
4	Department of Ophthalmology and Optometry, Medical University of Vienna	Austria	4	0	2021
5	Zhongshan Ophthalmic Center, Sun Yat-Sen University	China	4	0	2019
6	Casey Eye Institute, Oregon Health & Science University	USA	3	3	2021
7	College of Computer Science and Technology, Zhejiang University	China	3	3	2021
8	Department of Biomedical Engineering, Oregon Health & Science University	USA	3	3	2021
9	Department of Dermatology, Venerology and Allergology, University Hospital of Essen	Germany	3	3	2022
10	Department of Electronics and Computer Engineering, Ngee Ann Polytechnic	Singapore	3	0	2011

TLS, total link strength.

### 3.5 Bibliometric analysis of journals

**Figure 6:**
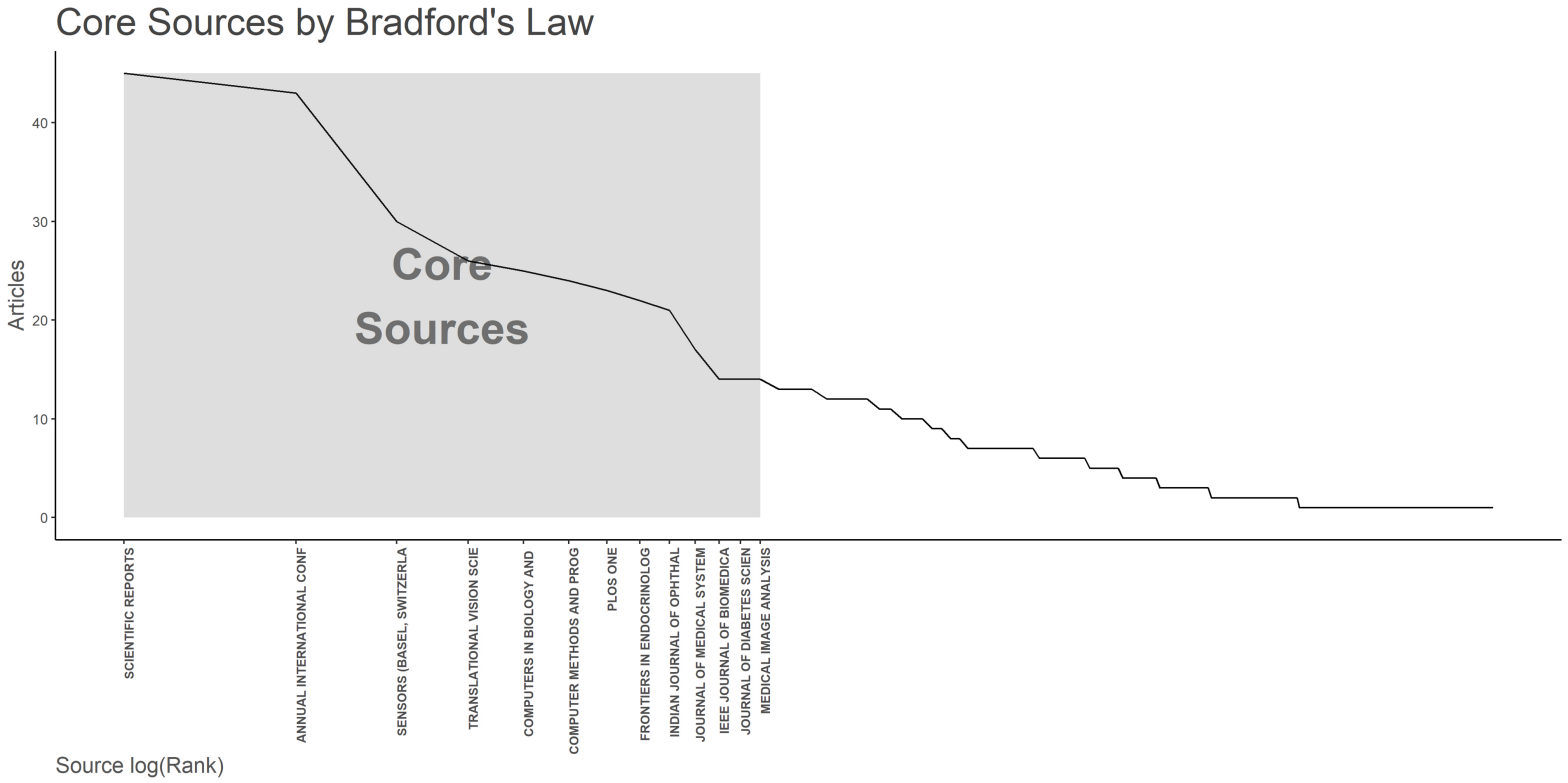
Core Journals by Bradford’s law. The x-axis represents the journal names, and the y-axis represents the number of publications. The gray area represents core journals, totaling 13.

A total of 249 different journals have published articles related to AI in diabetes complications. Thirteen core journals are shown in Figure 6. Table 4 presents detailed information on 13 core journals, including the number of publications, journal impact factor (JIF), h-index, and cite-score. Scientific Reports leads with 45 publications, followed by IEEE Transactions on Biomedical Engineering with 43 publications, and Sensors-Basel with 30 publications, ranking second and third, respectively. Medical Image Analysis has the highest JIF at 10.9, followed by Computers in Biology and Medicine and IEEE Journal of Biomedical and Health Informatics, both at 7.7. The journals with the highest h-index are PLoS One (332), Scientific Reports (213), and IEEE Transactions on Biomedical Engineering (200). Medical Image Analysis leads with a cite-score of 19.9, followed by IEEE Journal of Biomedical and Health Informatics (11.9) and Journal of Medical Systems (11.8).

**Table 4:** Top 13 core journals by publication productivity, journal impact factor, h-index, cite-score.

rank	Journals	Publications	JIF (2023)	H-index	Cite-score
1	Scientific Reports	45	4.6	213	7.5
2	IEEE Transactions on Biomedical Engineering	43	4.6	200	9.5
3	Sensors-Basel	30	3.9	172	6.8
4	Translational Vision Science & Technology	26	3.0	21	4.5
5	Computers in Biology and Medicine	25	7.7	94	9.2
6	Computer Methods and Programs in Biomedicine	24	6.1	102	10.1
7	PloS One	23	3.7	332	6.0
8	Frontiers in Endocrinology	22	5.2	68	5.6
9	Indian Journal of Ophthalmology	21	3.1	51	3.3
10	Journal of Medical Systems	17	5.3	79	11.8
11	IEEE Journal of Biomedical and Health Informatics	14	7.7	125	11.9
12	Journal of Diabetes Science and Technology	14	5.0	0	7.3
13	Medical Image Analysis	14	10.9	135	19.9

JIF, journal impact factor.

### 3.6 Bibliometric analysis of keywords

**Figure 7:**
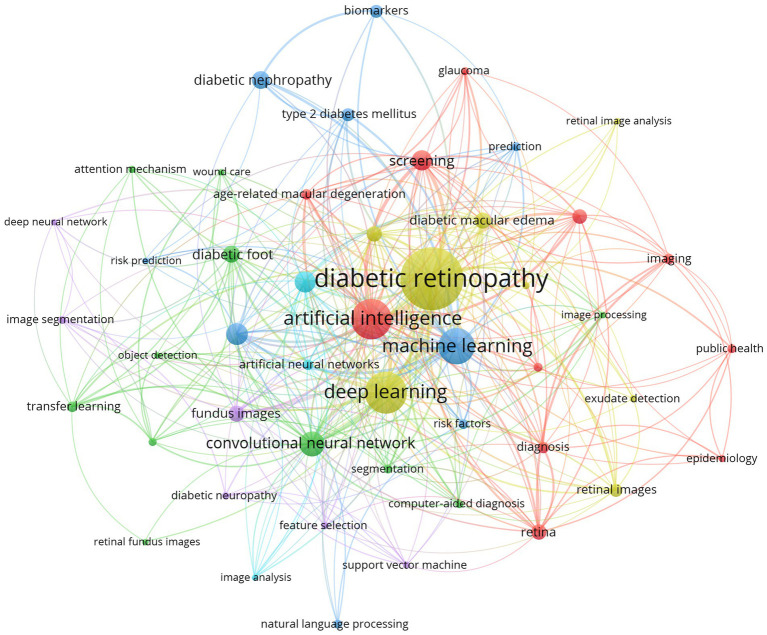
Author keywords co-occurrence network map. These 48 author keywords have been divided into 6 clusters with 341 links and a total link strength of 1,318. Each node represents an author keyword, and its size indicates the frequency of keyword occurrence. The lines connecting the nodes represent the cooccurrence relationship, and the thickness of the links represents the level of collaboration.

We conducted a co-occurrence analysis of author keywords for the retrieved 935 documents in Vosviewer, revealing a total of 1,182 keywords, of which 48 keywords appear more than 5 times. The visual network map of these 48 keywords is shown in Figure 7. The largest nodes in the graph are DR, followed by DL, AI, and ML, with DR and DL belonging to the yellow cluster, suggesting widespread application of DL in DR. Additionally, we selected the top 10 keywords based on frequency of occurrence, as shown in Table 5. The three most frequent diabetes complications are DR (Occurrences = 260, TLS = 457), DN (Occurrences = 28, TLS = 34), and DF (Occurrences = 26, TLS = 54). Notably, publications on DR have the earliest average publication year (2021), while those on the latter two have relatively later publication years (2022).

**Table 5:** The top 10 most frequent keywords.

Rank	Keywords	Occurrences	Links	TLS	Avg. pub. year
1	Diabetic retinopathy	260	44	457	2021
2	Deep learning	125	38	288	2021
3	Artificial intelligence	119	38	288	2022
4	Machine learning	100	33	193	2021
5	Convolutional neural network	53	28	121	2021
6	Diabetes mellitus	40	19	72	2021
7	Optical coherence tomography	38	21	89	2021
8	Screening	37	21	104	2022
9	Diabetic nephropathy	28	8	34	2022
10	Diabetic foot	26	15	54	2022

TLS, total link strength.

**Figure 8:**
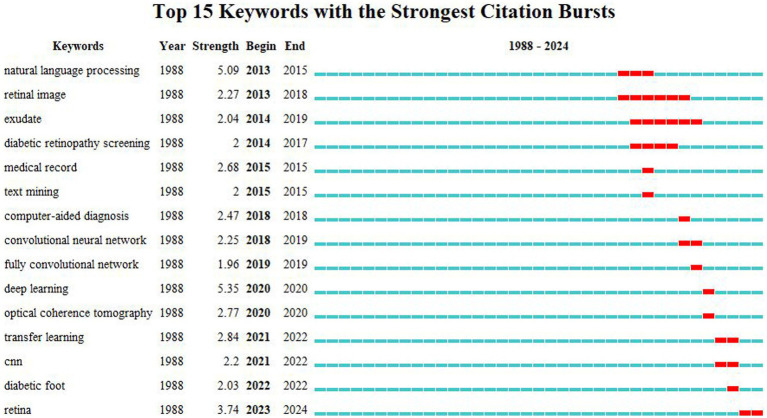
Top 15 keywords with the strongest citation bursts. The blue lines represent the base timeline, while the red segments represent the duration of the keyword burst, and the two endpoints correspond to the beginning and end times of the burst.

The top 15 keywords with the strongest burst in citations are shown in Figure 8. It can be observed that the field is continually developing and maturing over time. DL has the highest strength, mainly concentrated in 2020. Natural language processing and retina rank second and third, respectively, with natural language processing having the earliest appearance (2013–2015) and retina having the latest appearance (2023–2024). The remaining keywords have burst intensities below 3.0. Retinal image and exudate have the longest durations, spanning 5 years.

**Figure 9:**
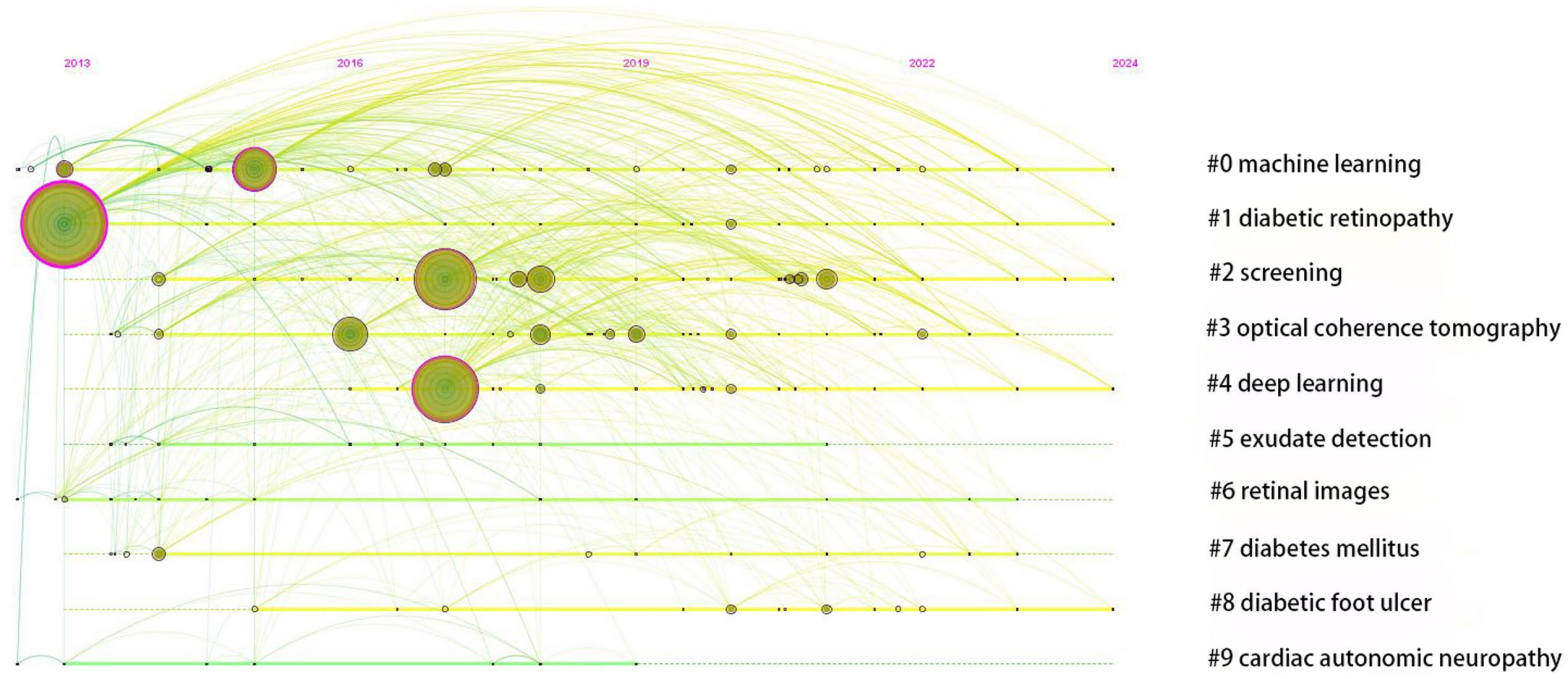
Timeline view based on cluster analysis. Each horizontal timeline represents a cluster, which is named after the most prominent node in each timeline, and nodes on the timeline represents keywords. The lines connecting the nodes represent the cooccurrence relationship, and the thickness of the links represents the level of collaboration. The timeline from left to right corresponds to 2013–202.

Furthermore, we conducted keyword timeline maps analysis for closely related and similar keywords in the field of AI in diabetes complications, as shown in Figure 9. Each horizontal line represents a cluster, and a total of 10 clusters have been researched since 2013. Clusters #0 ML, #1 DR, #2 screening, #4 DL, and #8 DF are still being researched in 2024, indicating that these topics are the focus of research in the field in the future.

## 4 Discussion

To the best of our knowledge, this study is the first comprehensive bibliometric analysis of the application of AI in the field of diabetes complications. Unlike focusing on diabetes, this study focuses on the serious diabetes complications, covering all AI technologies, providing researchers with a broader perspective.

### 4.1 General information

It can be observed that the research on AI in diabetes complications is becoming more and more extensive, and the number of published papers is increasing year by year, which is mainly due to the rapid development of emerging industries and the rapid progress of AI-assisted medical technology, which also highlights the huge potential of AI in healthcare applications. In this research field, the United States and China rank among the top in terms of the number of publications, international influence, and number of publishing institutions, which is closely related to the high level of science and technology development and the huge investment of the two countries. As one of the world’s leading countries in the development of AI, the United States has strong scientific and technological strength, educational background, investment environment and policy support in the field of AI. And for China, the huge population base is one of the key factors. At the same time, there are some differences in AI research on diabetes complications between the two countries. The United States focuses on precision medicine, technological innovation, and the development of high-end equipment, with an emphasis on developing decision support systems that can be applied clinically. For example, the United States has developed various AI-based decision support systems to optimize insulin dosage calculations and monitor diabetic complications (Li et al., 2020). In contrast, China places greater emphasis on the accessibility, cost-effectiveness, and coverage of primary healthcare services by AI, considering the practical needs of large-scale populations. For instance, Professor Huang Tianyin, in collaboration with teams from Shanghai Jiao Tong University and the National University of Singapore, has developed DeepDR-LLM, an integrated visual-large language model system, which can effectively improve DR screening and diabetes management at the grassroots level (Li et al., 2024). In the future, international cooperation should be further strengthened, combining large-scale data from China and cutting-edge technology from the United States to jointly promote the overall progress in the field of AI in diabetes complications (Sheng et al., 2024). In addition, the overall distribution of authors in this field is uneven, and the denser places form unique academic clusters, and the relationship between authors is not very close, which suggests that scholars in this field need more academic exchanges and closer academic cooperation in the future.

### 4.2 Hotspots and frontiers

#### 4.2.1 AI in DR

It is worth noting that the most rapid development of AI applications is in DR. On the one hand, this is made possible by the public Kaggle dataset (Kaggle, 2015) containing 100,000 retinal images. A number of algorithms have been developed by applying this dataset, which has given a huge boost to the development of AI. On the other hand, this is attributed to the increase in computer power and the application of CNNs and other DL techniques, which serve as assisting technologies capable of automatically screening DR from fundus photographs without manual input, exhibiting high sensitivity and specificity (>85%) (Rajesh et al., 2023). Our study also found that DR and DL are the two most frequently used terms and have the strongest citation bursts, further illustrating the high level of interest in both within this research area. Ahmed and Thrishulamurthy (2023) discussed the current research status of OCT imaging and CNNs integration for DR diagnosis, suggesting its transformative potential in DR diagnosis, facilitating early intervention, personalized treatment, and improving patient prognosis. By 2021, the FDA had approved the use of multiple AI-based DR screening systems for clinical application, such as IDx-DR and EyeArt (Abràmoff et al., 2018; Bhaskaranand et al., 2019). Furthermore, Dai et al. (2024) trained and validated a DL system named DeepDR Plus, using multi-ethnic datasets, demonstrating its capability to predict individualized risk and the time to DR progression within 5 years, thus enabling personalized screening intervals. Studies suggest that by 2030, AI algorithms will be applied in numerous large-scale DR screenings worldwide, either as fully autonomous systems or in hybrid systems where algorithms function as assistive tools (Xie et al., 2020). As AI image recognition algorithms become increasingly accurate, the diagnosis of DR will become more precise, significantly alleviating screening burdens.

#### 4.2.2 AI in DPN and DF

Early identification of risk factors for DPN and appropriate interventions can greatly delay the occurrence and development of DF. AI can help establish well-performing DPN risk prediction models to determine the most closely related risk factors for DPN. Lian et al. (2023) based on data from 1,278 diabetic patients, trained six ML models [logistic regression, k-nearest neighbor, decision tree, naive bayes, random forest (RF), and extreme gradient boosting (XGBoost)], finding that the XGBoost model outperformed others. Besides, AI is also crucial for DPN screening and diagnosis.

DF is a multifactorial severe complication more suitable for AI analysis and auxiliary diagnosis (Howard et al., 2023). To identify clinical and biochemical risk factors for DF, Nanda et al. (2022) analyzed patients with and without DF using various ML algorithms, discovering new risk factors for DF. They also proposed that the decision fusion strategy of the Stacking C algorithm improves prediction accuracy and can be used as a supplementary method for DF and its subtypes calculation. Moreover, Sharma et al. (2023) successfully developed a thermal DF dataset, effectively classifying the severity of DF using conventional ML and CNNs technologies, enhancing the detection and management of DF, and effectively improving patient prognosis. Although the current advanced ML algorithms can also detect, locate and segment the DF image to determine whether it contains diabetic foot ulcer, how to predict the occurrence of DF is a challenge. To do this, a large dataset of images annotated by specialists is needed for AI to learn and develop more advanced algorithms. Basiri et al. (2024) established a comprehensive dataset named Zivot, laying the foundation for further exploration of holistic and multimodal approaches to DF research. In addition, more and more researches are using AI to predict the risk of ulcers (Xiaoling et al., 2024; Hong et al., 2024), sepsis (Matsinhe et al., 2024) and amputation (Oei et al., 2024; Demirkol et al., 2024) in DF patients. In the future, more attention will continue to be paid to the diagnosis, classification, and prediction of DPN and DF, providing continuous health care for patients.

#### 4.2.3 AI in DN

Early detection of DN is crucial to prevent its progression to renal failure. By performing a keyword network analysis, we found that there is a strong connection between DN and biomarkers. Although some biomarkers for DN have been identified, none are sufficiently reliable for accurate diagnosis and prognosis prediction. To address this challenge, researchers have focused on identifying DN-related biomarkers and elucidating their pathogenic mechanisms, leading to the development of novel diagnostic and therapeutic approaches. At present, some valuable hub genes have been identified—FSTL1, CX3CR1, and AGR2 (Chen et al., 2024); VWF and DNASE1L3 (Dong et al., 2024); G6PC and HSD17B14 (Bi et al., 2024)— as innovative diagnostic biomarkers and therapeutic targets for DN by applying different AI methods. Furthermore, some researchers have utilized AI algorithms to predict the progression of DN in susceptible patients, enhancing early detection rates. Yin et al. (2024) demonstrated that XGBoost had the best performance in screening DN, highlighting the critical role of the least absolute shrinkage and selection operator (LASSO) in model selection accuracy and stability. In the future, with continued advancements in AI algorithms, researchers aim to develop more precise algorithmic models to aid in the discovery of new highly specific biomarkers, facilitating early diagnosis and treatment of DN.

#### 4.2.4 AI in other complications

In addition to the three most common diabetic complications discussed above, AI can also assist in diagnosing and treating other complications such as diabetic cardiomyopathies and DKA. Diabetic cardiomyopathy is one of the leading causes of increased mortality in diabetic patients. Predicting the progression of diabetic cardiomyopathy using ML algorithms combined with multiple cardiac biomarkers can facilitate early intervention (Segar et al., 2024; Irlik et al., 2024). DKA is a life-threatening but preventable acute diabetes complication. Both conventional ML (logistic regression and LASSO) and flexible ML (XGBoost, RF and feedforward network) methods can identify overlapping but distinct risk factors for DKA (Li et al., 2021). Future research on these complications will continue to focus on identifying more highly specific biomarkers, integrating AI for efficient identification, and intervening in treatment during the early stages of the disease, thereby significantly reducing mortality and disability rates for patients.

#### 4.2.5 Challenges in AI

At present, DL is one of the most widely used AI research methods in the field of diabetes complications, and commonly used models include: logistic regression, LASSO, k-nearest neighbor, decision tree, naive bayes, RF, and XGBoost. A commonly used type of data is unstructured data (images). For the dimensions of the data, the higher the dimension, the more features it contains, but at the same time, it will lead to a decrease in the performance of the model unless the sample size increases accordingly. The accuracy of a model depends primarily on the size of the training data and the quality of the algorithms employed. Although various databases currently exist [e.g., NCBI, EyePACS (Wang et al., 2024), Global Diabetes Atlas, DIARETDB1 (Kauppi et al., 2007), https://d2h2.maayanlab.cloud/, etc.], there are still some challenges to data quality and consistency. Firstly, missing values and noisy data. There may be missing values or measurement errors in diabetes complications data, reducing the accuracy of the models. Secondly, the data is not standardized enough. Data formats, recording methods, and indicator ranges may not be uniform across different medical institutions and devices, making it difficult to integrate and analyze data. Thirdly, there is a shortage of high-quality annotated data. Labeling medical data, such as lesion markers in imaging data, requires expert involvement, which is time-consuming and costly. Finally, due to patient privacy protection, factors such as racial, gender, and regional differences, and uneven disease distribution make it more difficult to obtain large-scale, high-quality data.

In terms of model performance and adaptability, it often faces problems such as insufficient generalization ability, overfitting risks, and complexity and interpretability of the models. AI algorithms are often considered “black boxes” (Chun and Kim, 2023) with opaque decision-making, making it difficult for doctors and patients to understand their decision-making logic. Based on this, new research has emerged in the direction of explainable AI. For example, Srinivasu PN et al. proposed an explainable diabetes prediction AI software system, XAI, which can effectively identify individuals with elevated blood glucose levels and explain expected outcomes and decision-making models (Srinivasu et al., 2024). Similarly, Hendawi R et al. developed the XAI4Diabetes framework, providing transparent and interpretable explanations for the diabetes prediction process and prediction outcomes, enhancing trust in AI predictions (Hendawi et al., 2023). However, more studies are needed on explainable deep learning for diabetes diagnosis with DeepNetX2. In the future, it is necessary to further strengthen the research in the direction of explainable AI to make its decision-making process more transparent and understandable.

In addition, ethics and fairness are another major challenge for AI. In AI research, it is necessary to place greater emphasis on data privacy protection and ethical issues, ensuring the security and confidentiality of patient data during storage and processing. At the same time, attention should be paid to whether AI systems have fully considered differences in race, gender, and economic status during design and training, so as to avoid exacerbating the unfair distribution of medical resources.

### 4.3 Limitations

Although AI is widely used in the diagnosis, treatment and prevention of diabetes complications and there are many literatures, there is a lack of systematic analysis. This study aims to address this gap by conducting a comprehensive and detailed bibliometric analysis of these publications using various analytical tools. However, it is important to acknowledge that there are still some limitations to this study. Firstly, we only selected the PubMed database for bibliometric analysis, which is popular in medicine and widely accepted for bibliometric analysis. However, there is a possibility that some studies were not included, potentially affecting the research findings. Secondly, we only selected articles in the English language category without analyzing or statistically evaluating articles in other language categories, introducing potential selection bias. Thirdly, citation analysis was not performed in this study as the data downloaded from the PubMed did not include citation data.

## 5 Conclusion

Over the past few decades, there has been a steady increase in the number of publications related to the research of AI in diabetes complications. The investigation of AI in DR and DF will be future research hotspots and frontier. This area of study can be further promoted by enhancing cooperation between countries, institutions, and authors. According to the current growth trend, it is expected that global research on AI applications in diabetes complications will be increased further. Future research will further focus on the clinical application of AI in the diagnosis, treatment, and prediction of diabetes complications. These findings can assist researchers in identifying future research directions and provide valuable insights and references for scholars.

## Data Availability

The original contributions presented in the study are included in the article/supplementary material, further inquiries can be directed to the corresponding author.
